# Attitudes towards people with mental illness among psychiatrists, psychiatric nurses, involved family members and the general population in a large city in Guangzhou, China

**DOI:** 10.1186/1752-4458-8-26

**Published:** 2014-07-03

**Authors:** Bin Sun, Ni Fan, Sha Nie, Minglin Zhang, Xini Huang, Hongbo He, Robert A Rosenheck

**Affiliations:** 1 Neuropsychiatric Research Institute, Guangzhou Psychiatric Hospital, The Affiliated Hospital of Guangzhou Medical College, Guangzhou, China; 2 Department of Nursing, Guangzhou Psychiatric Hospital, The Affiliated Hospital of Guangzhou Medical College, Guangzhou, China; 3 Department of Psychiatry, Yale University School of Medicine, New Haven, USA

**Keywords:** Survey, Mental Illness, Attitude

## Abstract

**Purpose:**

Stigma towards people with mental illness is believed to be widespread in low and middle income countries.

**Methods:**

This study assessed the attitudes towards people with mental illness among psychiatrists, psychiatric nurses, involved family members of patients in a psychiatric facility and the general public using a standard 43-item survey (N = 535). Exploratory factor analysis identified four distinctive attitudes which were then compared using Analysis of Covariance (ANCOVA) among the four groups, all with ties to the largest psychiatric facility in Guangzhou, China, adjusting for sociodemographic differences.

**Results:**

Four uncorrelated factors expressed preferences for 1) community-based treatment, social integration and a biopsychosocial model of causation, 2) direct personal relationships with people with mental illness, 3) a lack of fear and positive views of personal interactions with people with mental illness, 4) disbelief in superstitious explanations of mental illness. Statistically significant differences favored community-based treatment and biopsychosocial causation (factor 1) among professional groups (psychiatrists and nurses) as compared with family members and the general public (p < 0.001); while family members, unexpectedly, showed far weaker personal preferences for direct personal relationships with people with mental illness than all three other groups (p < 0.001).

**Conclusion:**

Both psychiatrists and nurses showed greater support for social integration and biopsychosocial understandings of mental illness than the lay public, most likely because of their training and experience, while family members showed the least positive attitudes towards direct personal relationships with people with mental illness. These findings suggest support for a more extensive, formal system of care that gives family members some distance from the problems of their relatives and support in their care.

## Introduction

Sigma and negative attitudes towards people with mental illness have been found to be common worldwide among both trained health professionals as well as the general population [1,2]. In China, mental illness not only refers to severe psychotic illness, but also contains more common disorders such as depressive disorders and anxiety disorders etc. Some studies have found attitudes toward mental illness were more stigmatized in less developed Asian and African cultures than in the West [3-5] but far less is known about differences in such attitudes between social groups within Lower Middle Income Countries (LMICs) such as China. For example some studies have focused on attitudes within groups and factors influencing such attitudes generally in China and Hong Kong [6,7] but no studies, to our knowledge, have examined differences in attitudes between professional groups, family members of people with mental illness, and the general public within a single community. The implementation of community-based mental health treatment and the achievement of social reintegration of people with mental illness in LMICs may depend on the development of positive attitudes towards this often stigmatized population within their families and the general public.

The Guangzhou Psychiatric Hospital (GPH) was the first psychiatric hospital in China [8] and is currently the largest psychiatric institution in Guangzhou city (population 13 million) and Guangdong province (population 100 million) and thus provides a useful setting in which to study variation in attitudes towards people with mental illness in a highly urbanized area of Southern China, and, perhaps, in China, more generally, the most populous LMIC in the world. The GPH hospital accounts for approximately 70% of all psychiatric beds in Guangzhou.

This study aimed to use a standard measure to compare the attitudes and beliefs towards people with mental illness along multiple empirical dimensions among different subgroups of with diverse connections to the GPH.

## Methods

This study sought to explore the perceptions about mental illness and the attitude toward the people with mental disorder among participating health care professional (physicians and nurses), patients’ family members and the general public with ties to the GPH.

### Sample

Physicians and nurses on the professional staff of GPH (N = 87 physician and 162 nurses) who work in both inpatient units and outpatient clinics were surveyed. Family members (N = 137) were invited to complete the survey while waiting for appointments for their kin at the GPH outpatient clinic, which predominantly provides services to former GPH inpatients. Representatives of the general population were social acquaintances of non-clinical professional staff of the GPH. For example, laboratory staff of GPH volunteered to invite a convenience sample of acquaintances (friends, social acquaintances and their families with no professional mental health background to complete the survey (N = 149). The period of data collection was from January to May in 2012. Waiver of written informed consent was provided by the Institutional Review Board of GPH.

### Measures

The questionnaire was a modified version of FABI (Fear and Behavioral Intentions toward the mentally ill) [2] with additional items derived from the CAMI (Community Attitudes to Mental Illness) [9] developed by Taylor and Dear and from a modified version of a questionnaire developed for the World Psychiatric Association Program on stigma and mental illness [10]. The questionnaire included 43 dichotomous (yes/no) questions with some supplemental items concerning the impact witchcraft and curses developed for use in West Africa [11,12] and supplemental items documenting respondent socio-demographic characteristics.

The socio-demographic questions addressed age (measured in years), gender, years of education, marital status (not married vs. married), area where born (urban, semi-urban, rural), area of current residence (urban, semi-urban, rural), current medical staff status (health professional staff (physicians vs. nurses), patient relative status or others (friends and acquaintances of non-professional staff).

### Analysis

Exploratory factor analysis (see details below) was used to identify items that reflected common domains. Items that were negatively worded were re-coded in a positive direction for consistency (e.g. Witchcraft is a cause of mental illness was recoded in the direction to suggest that superstitions like witchcraft are *not* believed to be a cause of mental illness). Weighted factor scores were computed giving greater weight to items that load more strongly on each factor.

Chi square tests and analysis of variance were used to identify differences between the four groups (physicians, nurses, family members, members of the general public) on potentially confounding sociodemographic characteristics. Analysis of Covariance was then conducted to compare estimated mean scores on each attitudinal domain adjusting for potentially confounding sociodemographic characteristics.

Exploratory factor analysis with orthogonal matrix rotation was carried out to optimize independence and interpretability of factors in this sample and to identify latent relationships between the variables. A factor analysis with Varimax solution yields results which make it as easy as possible to identify each variable with a single factor. Altogether 36 items having factor weights greater than 0.4 were retained and 4 factors were identified. Factor scores were calculated based on item weights such that a one unit difference in factor scores represents a one-standard deviation difference on each factor.

All analyses were performed using SPSS13.0. Statistical significance was evaluated at the 0.008 level for 6 paired comparisons between the four groups (0.05/6 = 0.008).

## Results

### Sample

The sample included 535 respondents: 249 clinical providers (79 physicians [16.2% of the total sample] and 162 nurses [30.3% of the sample), and 286 non-clinicians (137 patient relatives [25.6% of the sample] and 149 friends or acquaintances of GPH non-clinical staff [27.9% of the sample]). Sociodemographic characteristics were significantly different across the four groups (Table 1). Of the 535 participants, 246 (46%) were male, 290 (54.2%) were with a mean age of of 33.8 years (SD = 11.5) and means years of education of 15.1 years (SD = 3.6).

**Table 1:** Chi square and analysis of variance comparison of group characteristics (N = 535)

**Demography variables**	**Psychiatrists (N = 87, 16.3%)**	**Nurses (N = 162, 30.3%)**	**Family members (N = 137, 25.6%)**	**General public (N = 149, 27.9%)**	**Total (N = 535,100%)**	**Statistics**	***P*-value**
**Age**	34.2 ± 8.6	29.6 ± 6.5	43.2 ± 14.1	29.2 ± 9.2	33.8 ± 11.6	*F* = 59.0	<0.001
**Gender**						*χ*^*2*^ = 28.8	<0.001
**Male**	48(55.2%)	47(29%)	67(49.3%)	84(56.4%)	246(46%)		
**Female**	39(44.8%)	115(71%)	69(50.7%)	65(43.6%)	288(53.8%)		
**Missing N**					1(0.2%)		
**Marital status**						*χ*^*2*^ = 51.0	<0.001
**Single**	26(30.2%)	74(46%)	37(27.6%)	93(66.9%)	230(43%)		
**Married**	60(69.8%)	87(54%)	97(72.4%)	46(33.1%)	290(54.2%)		
**Missing N**					15(2.8%)		
**Education years**	17.9 ± 2.5	15.9 ± 2.7	11.7 ± 3.2	15.4 ± 3.0	15.1 ± 3.6	*F* = 89.3	<0.001
**Birthplace**						*χ*^*2*^ = 51.0	<0.001
**Urban area**	39(44.8%)	63(39.4%)	69(50.7%)	38(25.5%)	209(39.1%)		
**Semi-urban area**	17(19.5%)	21(13.1%)	29(21.3%)	29(19.5%)	96(17.9%)		
**Rural area**	31(35.6%)	76(47.5%)	38(27.9%)	82(55%)	227(42.4%)		
**Missing N**					3(0.6%)		
**Place of residence**						*χ*^*2*^ = 51.0	<0.001
**Urban area**	79(92.9%)	131(81.9%)	80(59.3%)	100(67.1%)	390(72.9%)		
**Semi-urban area**	2(2.4%)	16(10%)	38(28.1%)	28(18.8%)	84(15.7%)		
**Rural area**	4(4.7%	13(8.1%	17(12.6%	21(14.1%	55(10.3%		
**Missing N**					6(1.1%)		

### Factor Structure

Inspection of the scree plot suggested a four factor solution (the first 8 eigenvalues are 8.988, 3.905, 2.983, 2.008, 1.430, 1.202, 1.116, and 1.058 respectively). The four factors, presented with individual item weights in Table 2, were interpreted as favoring 1) community-based treatment, social integration and a biopsychosocial model of causation, 2) personal preferences for direct personal relationships with people with mental illness, 3) a fear-free and positive view of specific interactions with people with mental illness, 4) disbelief in superstitious explanations of mental illness (e.g. witchcraft, curses).

**Table 2:** Four factor item weights

**ITEMS (paraphrased)**	**Weights**
**Factor1 Community treatment and Biopsychosocial causation**	
**Increased spending on mental health services is not a waste of money.**	**0.850**
**People with mental illness deserve our sympathy.**	**0.845**
**We have a responsibility to provide the best possible care for people with mental illness.**	**0.790**
**No-one has the right to exclude people with mental illness from their neighborhood.**	**0.790**
**Locating mental health facilities in a residential area doesn't downgrade the neighborhood.**	**0.785**
**We need to adopt a far more tolerant attitude toward people with mental illness in our society.**	**0.760**
**Anyone with mental illness should be given responsibility.**	**0.733**
**People with mental health problems should have the same rights to a job as anyone else.**	**0.722**
**Virtually anyone can become mentally ill.**	**0.706**
**Residents have nothing to fear from people coming to their neighborhood to obtain mental health services.**	**0.684**
**Mental health services should be provided through community based facilities as far as possible.**	**0.650**
**The best therapy for many people with mental illness is to be part of a normal community.**	**0.535**
**It is not frightening to think of people with mental problems living in residential neighborhoods.**	**0.524**
**Mental hospitals are an outdated means of treating people with mental illness.**	**-0.562**
**Traumatic event or shock can cause mental illness**	**0.500**
**Biological factors (other than brain disease or genetics) can cause mental illness.**	**0.490**
**Drug or Alcohol misuse can cause mental illness.**	**0.462**
**Genetic inheritance can cause mental illness.**	**0.437**
**Factor2 Socializing**	
**If somebody had been a former psychiatric patient, I would have them as a friend.**	**0.603**
**If somebody who had been a former patient came to live next door to me, I would visit them.**	**0.518**
**In interacting with someone with mental illness, I could maintain a friendship.**	**0.514**
**I would invite somebody who suffered from mental illness into my home.**	**0.490**
**I would occasionally greet somebody who had been a former patient and came to live next door to me.**	**0.455**
**I would not object to having mentally ill people living in my neighborhood.**	**0.437**
**I would have casual conversations with neighbors who had suffered from mental illness.**	**0.423**
**I would be willing to work with somebody with a mental illness.**	**0.422**
**Factor3 Specific interactions**	
**In interacting with someone with mental illness, you were not upset or disturbed about working on the same job.**	**0.471**
**Physical abuse cannot cause mental illness.**	**0.465**
**People with mental illness are not a public nuisance.**	**0.426**
**People with mental illness are not dangerous because of violent behavior.**	**0.423**
**In interacting with someone with mental illness, I would not be unwilling to share a room.**	**0.414**
**You would not avoid conversations with neighbors who had suffered from mental illness.**	**0.404**
**Factor4 Disbelief in witchcraft**	
**God's punishment cannot cause mental illness.**	**0.652**
**Someone puts a curse on you cannot cause mental illness.**	**0.569**
**Witchcraft cannot cause mental illness.**	**0.555**
**Possession by evil spirits cannot cause mental illness.**	**0.543**

High internal consistency reliability (Cronbach’s alpha) was found for factors 1, 2 and 4 (alpha 0.89, 0.78, 0.70 respectively) with a less satisfactory value for factor3 (alpha = 0.52).

### Group comparisons

**Figure 1:**
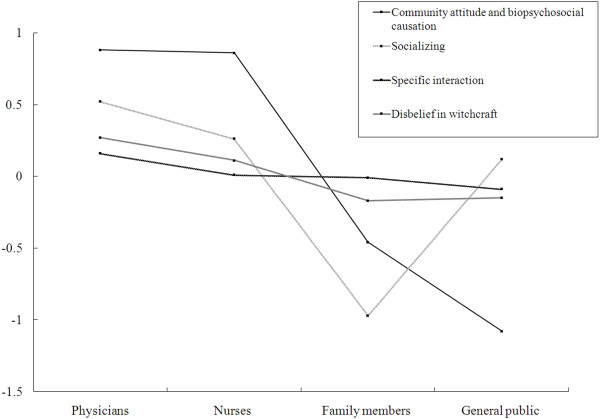
The mean standardized factor scores of four groups in each factor.

Paired comparison of mean values on factor 1 (community-based treatment, social integration and a biopsychosocial model of causation) (Table 3, Figure 1) showed no significant differences between psychiatrists and nurses both of whom scored significantly higher than family members (with a large effect size of almost 1.5) who, in turn, scored higher than members of the general public, (with a more modest effect size of about 0.5) after adjustment for differences in sociodemographic characteristics.

**Table 3:** ANCOVA and paired comparison of factor score across the four groups

**Factors**	**Means (SD) of factor scores**				***P*-value**	**Paired comparison**
	**Physician (Group1)**	**Nurses (Group2)**	**Family members (Group3)**	**General public (Group4)**		
**Community attitude and biopsychosocial causation**	0.88(0.37)	0.86(0.39)	-0.46(0.74)	-1.08(0.41)	<0.001**	Group: 1, 2 > 3 > 4
**Socializing**	0.52(0.32)	0.26(0.70)	-0.97(1.18)	0.12(0.93)	<0.001**	Group:1, 2, 4 > 3
**Specific interaction**	0.16(0.99)	0.01(0.98)	-0.01(1.09)	-0.09(0.96)	0.964	
**Disbelief in witchcraft**	0.27(0.95)	0.11(0.91)	-0.17(1.05)	-0.15(1.04)	0.079	

**P < 0.01.

ANCOVA of factor 2 (2) preferences for close, direct personal relationships with people with mental illness), (Table 3, Figure 1) showed that family members’ scores was markedly lower than physicians, nurses and general public, with large effect size differences of 1.1-1.5). There were no significant differences, in contrast, between physicians, nurses and the general public.

ANCOVA of factors 3 and 4 show no statistically significant group differences.

## Discussion

This study surveyed attitudes towards people with mental illness among clinicians (psychiatrists and nurses) and lay persons (family members and linked non-professionals) associated with GPH, the largest psychiatric hospital in Southern China i.e. China, South of the Yangtze River. Factor analysis of a standard 43-item survey identified four factors representing uncorrelated attitudes towards people with mental illness. Significant group differences, adjusting for sociodemographic characteristics were found on two of the factors.

First, psychiatrists and nurses more strongly endorsed the factor reflecting attitudes favoring community-based treatment, social integration of people with mental illness and a biopsychosocial model of causation than non-clinicians, including both family members and representatives of the general public. The effect size of this difference was large representing 1.5-2 standard deviations and most likely reflects their professional training and experience.

In addition family members endorsed such attitudes more strongly than friends and acquaintances of non-clinical hospital staff, albeit with a smaller effect size difference of about 0.5 standard deviations. It thus appears that professionals involved in the delivery of hospital-based psychiatric care strongly favor a shift to a more community-based system of care, one that might be quite different from the one hospital-based system in which they currently work. The linkage of such attitudes with a biopsychosocial view of the causation of mental illness can be taken as suggesting that this view of the etiology of mental illness may be part of a world-wide professional culture in which a medical model of mental illness, albeit one that also recognizes the importance of psychosocial factors, is viewed as supporting community-based models of care. One may infer from these data that in the view of mental health professionals, more than lay people, and current treatment system can and should allow the vast majority of patients to be cared for in non-institutional settings. In China in 2012 a new mental health law was enacted which fosters voluntary as contrasted with involuntary hospitalization and community based treatment. Nevertheless community psychiatric treatment and services are still in their infancy in China, and the vast majority of mental health services are still provided in in-patient settings or hospital-based out-patient departments, and many patients with mental illness are virtually confined at home or in the hospital for extensive periods of time [13]. It is also notable that family members of outpatients being treated at GPH endorsed this view somewhat more strongly than representatives of the general community with some connection to the hospital. Since neither professionals nor family members have experienced such a system of care directly, their shared values along these lines, and their difference in attitudes from representatives of the general public, may reflect developing progressive trends in contemporary professional ideology that has extended to family members who are involved in the developing culture of mental health service delivery in China.

In contrast to the findings for the first factor, family members had distinctively different attitudes that the other three groups on the second factor (2) preferences for direct personal relationships with people with mental illness. While these may seem to be contradictory findings, careful examination of the items in factor one show that they represent abstract principles that do not touch on the respondent’s personal conduct or more intimate dealings with people who have had a mental illness. Factor two, in contrast, addresses more direct individual interactions such as having someone as a friend, visiting with them in their home, inviting them into one’s own home, or having them as co-workers or neighbors. The contents of this factor are similar to social distance scales which can be measured as a unidimensional component of stigma towards people with mental illness [14]. The result agree with what others [15,16] had found -- that professionals with knowledge of mental disorders tolerate less social distance with patients with mental illness. While some have suggested that contact with people with mental illness is associated with less social distance [17-19], our results indicate that at least in China, where families must should much of the burden of mental illness alone, family member showed preference for more social distance than the general public. That family members of patients are the only group that participated in the survey that actually has such contact with patients and that they shoulder almost complete responsibility for them, may explain why they seem to find the prospect of such contacts far less appealing than those who are able to, maintain professional distance or have little direct contacts with people with mental illness at all, and hat.

The implications of these finding for practice and policy are complex. In many LMIC countries, families take primary responsibility for taking care of mentally ill relatives, and provide most of their care directly in their homes, with little assistance from public services. In a study from Nigeria [20], poorer social support was found to be associated with higher family burden. Natasha [21] report that in a sample from India, that family members who lack social support more frequently experience poor quality of life. Chinese family members must take responsibility for with patients with mental illness experience poorer functioning and less social support along with a higher level of caregiver burden and these stresses are likely reflect in their responses to questions about their intimate relationsships with people with mental illness [22]. Family members in LMIC countries seem to want to see the development of more progressive systems of community-based care, but in the absence of such systems, people from many countries have been candid in expressing their reluctant attitude towards close intimate relationships with people with mental illness, perhaps reflecting the stress they experience in their relationships with their own relatives. This finding should not be taken as a rejection of the model of community-based mental health care, but rather provides strong grounds for supporting the develop a more supportive, formal system of care that gives family members some distance from the problems of their relatives. This result draws attention to the crucial issue of what has been called "family burden" the painful, often debilitating and health endangering experience of caring for seriously mentally ill family members’ day in and day out. These experiences may be especially strong in Asian countries such as China in which the family, rather than the state or society, has a heavy burden of responsibility for mentally ill relatives, extending even to fiscal responsibility for their occasional damage of neighborhood property or criminal conduct [23,24].

On the final two factors, no significant differences were observed between the groups. The third factor (a fear-free and positive view of specific interactions with people with mental illness) addresses, for the most part, abstract and high moral principles involving human rights and privileges that would attract wide agreement, although several items mention intimate associations such as sharing a room or engaging in conversation.

The final factor (Additional file 1), reflecting disbelief in superstitious explanations of mental illness such as witchcraft or curses, not surprisingly, reflects widely shared secular humanistic values, characteristic of popular modern Chinese culture. To the extent that religious faiths have gained membership under recent reforms in China in recent decades, they predominate in rural areas quite different from heavily urbanized areas like Guangzhou, and bear few vestiges of superstitious beliefs that may have characterized dominant faiths of past centuries.

Several methodological limitations require comment. First, structured surveys offer only an imperfect approach to identifying subtle attitudes since they require simple responses to pre-structured questions, in this instance, all of which called for simple "yes-no" answers. Second the survey instrument used here, while previously used is several studies [25-27] has not been subject to independent validation using either quantitative or qualitative methods. Thirdly the interpretation of factor analysis can be challenging as items that are correlated statistically, do not always identify clearly homogeneous themes. Our first factor combined favorable attitudes towards community mental health with support for a biopsychosocial model of the etiology of mental illness. While these themes are somewhat different they usefully point to the close relationship between progressive thinking about the cause of mental illness and positive attitudes towards humane community-based care. Fourthly these results reflect the responses of only a few hundred respondents linked in diverse ways to one large psychiatric hospital in one of China’s largest cities. The generalizability of these results to other areas of China or to other LMICs is unknown. Finally attitude surveys do not necessarily predict or correlate with behavior [28] which may be shaped as much or more by economic resources as by personal attitudes and may provide limited predictive power or normative guidance for clinical practice or public policy.

Nevertheless this is one of the first surveys to compare attitudes among diverse but salient stakeholders with interests in the development of community mental health services in LMIC countries and as such has identified differences in attitudes across groups that will hopefully support, even in their diversity, the future development of community health care in LMICs such as China.

## Ethic Statement

This study is part of the project "investigation of risk factors of re-hospitalization for patients with mental illness" supported from funding from Chinese National Key Clinical Program in Psychiatry to Guangzhou Psychiatric Hospital, Guangzhou, China. The project was approved by the Ethic Committee of Guangzhou Psychiatric Hospital. This part of survey with WPA questionnaires was waivered of written informed consent.

## Competing interest

All authors have no interests to disclose.

## Authors’ contributions

HH and RR designed the study, supervised the data collection, and assisted with writing the article. BS wrote the paper and carried out the statistical analysis. SN, MZ and XH acquired the data and NF assisted with writing the article. All authors read and approved the final manuscript.

## Supplementary Material

Additional file 1 **The endorsement rates of each items of *Disbelief in witchcraft* among four groups.** Click here for file
