# Supplementary material for: Attitudes towards people with mental illness among psychiatrists, psychiatric nurses, involved family members and the general population in a large city in Guangzhou, China
Source: Int J Ment Health Syst. 2014 Jul 3;8:26. doi: 10.1186/1752-4458-8-26 (PMC4105504; doi:10.1186/1752-4458-8-26)
Supplement: Additional file 1 — The endorsement rates of each items of Disbelief in witchcraft among four groups. [file 1752-4458-8-26-S1.doc]

Additional file 1**.** The endorsement rates of each items of ***Disbelief in witchcraft*** among four groups

| **The items of Disbelief in witchcraft** | **Psychiatrists (N=87)** | **Nurses (N=162)** | **Family members (N=137)** | **General public (N=149)** |
| --- | --- | --- | --- | --- |
| **God's punishment cannot cause mental illness.** | 89.7% | 81.3% | 87.8% | 92.6% |
| **Someone puts a curse on you cannot cause mental illness.** | 89.7% | 86.4% | 85.1% | 88.6% |
| **Witchcraft cannot cause mental illness.** | 90.9% | 76.3% | 84.1% | 86.4% |
| **Possession by evil spirits cannot cause mental illness.** | 81.6% | 76.4% | 73.6% | 87.9% |
